# A Review of Quantitative Tools Used to Assess the Epidemiology of Porcine Reproductive and Respiratory Syndrome in U.S. Swine Farms Using Dr. Morrison’s Swine Health Monitoring Program Data

**DOI:** 10.3389/fvets.2017.00094

**Published:** 2017-06-27

**Authors:** Carles Vilalta, Andreia G. Arruda, Steven J. P. Tousignant, Pablo Valdes-Donoso, Petra Muellner, Ulrich Muellner, Moh A. Alkhamis, Robert B. Morrison, Andres M. Perez

**Affiliations:** ^1^Department of Veterinary Population Medicine, College of Veterinary Medicine, University of Minnesota, St. Paul, MN, United States; ^2^Department of Preventive Veterinary Medicine, College of Veterinary Medicine, The Ohio State University, Columbus, OH, United States; ^3^Swine Vet Center PA, St. Peter, MN, United States; ^4^Boehringer Ingelheim Animal Health, St. Joseph, MO, United States; ^5^Department of Agriculture and Resource Economics, University of California, Davis, Davis, CA, United States; ^6^Epi-interactive, Wellington, New Zealand; ^7^Environment and Life Sciences Research Center, Kuwait Institute for Scientific Research, Kuwait City, Kuwait

**Keywords:** porcine reproductive and respiratory syndrome, Swine Health Monitoring Project, data sharing, epidemiology, spatiotemporal analysis

## Abstract

Porcine reproductive and respiratory syndrome (PRRS) causes far-reaching financial losses to infected countries and regions, including the U.S. The Dr. Morrison’s Swine Health Monitoring Program (MSHMP) is a voluntary initiative in which producers and veterinarians share sow farm PRRS status weekly to contribute to the understanding, in quantitative terms, of PRRS epidemiological dynamics and, ultimately, to support its control in the U.S. Here, we offer a review of a variety of analytic tools that were applied to MSHMP data to assess disease dynamics in quantitative terms to support the decision-making process for veterinarians and producers. Use of those methods has helped the U.S. swine industry to quantify the cyclical patterns of PRRS, to describe the impact that emerging pathogens has had on that pattern, to identify the nature and extent at which environmental factors (e.g., precipitation or land cover) influence PRRS risk, to identify PRRS virus emerging strains, and to assess the influence that voluntary reporting has on disease control. Results from the numerous studies reviewed here provide important insights into PRRS epidemiology that help to create the foundations for a near real-time prediction of disease risk, and, ultimately, will contribute to support the prevention and control of, arguably, one of the most devastating diseases affecting the North American swine industry. The review also demonstrates how different approaches to analyze and visualize the data may help to add value to the routine collection of surveillance data and support infectious animal disease control.

## Introduction

The U.S. is home to one of the largest swine industries worldwide; although the country is one of the largest pork exporters globally, the domestic market is still the most important for the industry, with 112 million hogs processed in national slaughterhouses in 2013 and with an average breeding stock of 6 million sows in the last 10 years (1–3). Approximately 50% of the hog production is concentrated in three states, namely, Iowa, North Carolina, and Minnesota (4). The concentration of such a large population in a relatively small area resulted in high density, and consequently, relatively high vulnerability of the industry to the introduction and spread of infectious diseases. The industry has tried to mitigate this vulnerability by increasing biosecurity and other prevention strategies, including air filtration and quarantine of incoming pigs (5, 6).

Porcine reproductive and respiratory syndrome (PRRS) is, arguably, one of the most economically devastating diseases affecting the U.S. swine industry, causing annual losses estimated to be between $550 and $664 million (7, 8). The disease, caused by the infection of an *Arterivirus*, referred to as PRRS virus (PRRSv), emerged in the U.S. in the late 1980s and, since then, has remained prevalent in the country (9, 10). PRRSv is an RNA virus, which has led to the appearance of a wide array of emerging or reemerging strains with a large variety of phenotypic and genotypic features (11). Commercial vaccines do not prevent infection although they are reported to reduce virus shedding and decrease clinical impact. Protection is reported to be more profound after infection with homologous than heterologous strains (12, 13). Modified live vaccines are more effective than those formulated with inactivated virus (14). Many alternative mechanisms of transmission have been described for PRRSv, including direct transmission through the movement of pigs and indirect through fomites, insects, semen, or airborne spread (6, 15–20). The American Association of Swine Veterinarians (AASV) developed a classification system with four categories based on infection status in sow herds. A herd was defined as “unstable” if field virus could be detected within the herd. These categories are referred to as positive unstable (Category I), positive stable (Category II), provisional negative (Category III), and negative (Category IV) (21). In the absence of a regulatory framework, data on farm-level infection incidence can only be collected on a voluntary basis in the U.S.

The University of Minnesota started a voluntary project in 2011 referred to as the Dr. Morrison’s Swine Health Monitoring Program (MSHMP) with two main objectives, namely, (1) to, in the short term, translate shared data into knowledge that informs veterinarian and producer decisions and (2) to, in the long term, build capacity to respond, in an effective and timely manner, to emerging and reemerging pathogens that may threaten the swine industry. Initially, participants shared weekly PRRS status data for sow farms, following and adapting AASV guidelines (21). As the program progressed, the MSHMP expanded, and some participants are also sharing data for porcine epidemic diarrhea (PED) and porcine delta corona virus. The MSHMP is quite unique because, despite its voluntary nature, it currently receives weekly data from sow farms corresponding to approximately 45% (*n* = 2,854,000) of the sows in the country according to the U.S. Census (3), and for that reason, it is, arguably, one of the largest datasets of voluntarily shared information by producers on swine health in the world. The MSHMP, therefore, demonstrates how epidemiological knowledge on one of the most pressing diseases for a large industry may be gained through the analysis of routinely collected data, as part of a private–academic partnership and in the absence of a regulatory framework.

The objective of the paper here was to summarize the methods and results of a variety of analytic tools applied to MSHMP data to elucidate the epidemiological dynamics of PRRS in the U.S. This review provides a summary of knowledge gained over the last years on PRRS dynamics through the use of different types of analytical methods for the analysis of routinely collected surveillance data. In addition, the review will ultimately contribute to demonstrate the application of epidemiological analytic tools in supporting the prevention and control of, arguably, one of the most economically challenging diseases for the swine industry worldwide.

## Materials and Methods

A systematic pathway was used to direct the application of epidemiological tools to assess the epidemiological dynamics of PRRS and its related components, as described elsewhere (22).

### Space and Time Clustering

Two different approaches and tools were used to quantify the spatial dependence of PRRS incidence since 2011. Specifically,

- Spatial scan statistical models were used to determine the repeatability of annual patterns of disease incidence in space and to compare the spatial distribution of the disease before and after the country was affected by PED (23, 24). An exponentially weighted moving average and an upper confidence limit were used to signal the onset of the epidemic season.
- Space and time point process assessed by G- and K-functions were used to quantify the clustering of PRRS outbreaks, as well as data sharing to evaluate its relationship with PRRS incidence on a subset of participants (25).

### Factors That Influence PRRS Risk

- The significance of quarterly and annual PRRS incidence from 2009 to 2013 was assessed using a chi-square test. Furthermore, logistic regression was used to estimate the likelihood of the presence of PRRSv infection in a given year increasing the odds of that herd being infected the following year (23). Chi-square test and logistic regression models were also used to compare PRRS incidence between 2009–2012 and 2013, the year that PED was first detected in the U.S., and used to explain how its emergence might have affected the temporal dynamics, and overall incidence of PRRS (24).
- A general linear mixed-effects logistic regression model was used to investigate the association between factors hypothesized to influence PRRS risk (i.e., month, farm type, county density, active participation in a voluntary control program, and proportion of farms stable) and PRRS incidence (25).
- A mixed-effect Poisson regression model was built to evaluate the effects of land coverage (e.g., cultivated fields and shrubs or trees) and geographical features (including terrain slope and altitude) on the incidence of PRRS outbreaks considering the years of 2009–2016. The model included a random effect at the production system level and also included explanatory variables that were known to affect the risk of PRRS outbreaks such as pig density, farm size, and geographical region (26).
- Species distribution models, such as presence-only maximum entropy machine learning algorithm, were used to build risk maps through predicting the spatial distribution of PRRSv outbreaks using a set of environmental variables.^1^ That algorithm can extract associations to characterize the demographic and climatic requirements for PRRSv, and subsequently deploying those associations to predict suitable geographical locations in non-sampled areas, where most likely PRRSv outbreaks will be detected (see text footnote 1).

### Evolutionary Analysis

A large dataset (*n* = 6,774) of open reading frame 5 (ORF5) complete sequences provided by five production systems was used to study the evolution and spread of PRRSv across included systems. Bayesian phylogenetic analysis with divergence time, growth rate, population size, and estimation of viral dispersal history between systems was applied to this dataset to understand the order of events and spatiotemporal progression of this specific cluster (27).

### Modeling

Time-dependent reproduction (TD-R) numbers for PRRS across different regions and production systems within the U.S. were estimated. The uses of TD-R include monitoring of epidemic trends over time, identification of “super-spreader events,” measurement of progress of interventions over time, and extraction of parameters for mathematical models (e.g., models to test interventions) (28).

### Prototype of a Real-time Data Visualization Dashboard

Finally, an application was developed in RStudio Shiny (29) to allow for real-time visualization of MSHMP data including analytical outputs of different types of analysis [Pig/Statistical Analysis and Visual Insights (SAVI)]. Pig/SAVI allows for data visualization and analysis using web-based and standalone versions. JavaScript and CSS were used to enable some of the functionality and visual elements. Several functional elements were utilized, including reactivity, isolation, and download of outputs into CSV format.

## Results

### Space and Time Clustering

Porcine reproductive and respiratory syndrome showed a repetitive pattern within a cohort of sow farms in time and space. It was determined that the onset of the PRRS season began every year during the fall (between the months of October and November). Furthermore, spatial clusters of PRRS positive farms were consistently identified in similar locations throughout the study period (23, 24). Spatial trends in PRRS case distribution after PED emergence were similar to those found in previous studies at the national level (23, 24), although PRRS incidence decreased during that year. These data suggest that changes in management and biosecurity to avoid spread of PED may have affected PRRS incidence nationally without affecting the spatial dependence of PRRS incidence.

### Factors That Influence PRRS Risk

Farms that were PRRS infected in a given year were more likely to be infected in consecutive years compared to those that were not affected by PRRS. However, during the year in which PED emerged in the U.S., that pattern changed and the described association could no longer be observed. Furthermore, PRRS incidence was significantly lower, and the beginning of the PRRS season was delayed, compared to previous years (23, 24).

Analysis of data collected on a regional project in Minnesota, referred to as RCP-N212, suggested an increased interest of producers in participating in regional control programs during the study period, from July 2012 to June 2014. That analysis also showed, however, that participation on a regional project is not necessarily highly correlated with probability of data sharing. Time within the analyzed period (24 months) and the probability of sharing PRRS status to the voluntary regional control program were negatively associated with disease outbreaks, while density (as number of farms per square mile) was positively associated with PRRS outbreaks. Findings in this study showed that despite a statistical decreasing trend of the disease, spatiotemporal clustering of PRRS was observed along the whole study period. In turn, farm type (i.e., farms with and without breeding herds) did not show a specific association with PRRS, although farms with sows were more prone to report PRRS status in the RCP-N212 (25).

Land-related variables and land coverage appeared to have an impact on the incidence of PRRS outbreaks. Sow farms located in areas of cultivated/managed areas had higher incidence of PRRS outbreaks when compared to sow farms located in areas characterized by having herbaceous areas or trees. Altitude, as expressed in meters above sea level, did not appear to be associated with the occurrence of PRRS outbreaks; however, farms in slopes larger than 9%, when compared with the neighbor, had lower incidence of PRRS outbreaks compared to those located in areas characterized by slopes less than 2% (26).

Results of the maximum entropy model suggested that hog density was the most important predictor for PRRS incidence, whereas a number of environmental factors accounted for much of the remaining risk. More specifically, when considering different regions, swine density still has a role in impacting the number of cases being a driven factor in swine denser areas. However, some other variables such as precipitation or temperature can have an impact on the spread of the disease (see text footnote 1).

### Evolutionary Analysis

Spatial distribution of farms and production systems has an important role in the maintenance of endemic PRRSv strains and the occurrence of epidemic PRRS episodes. Recent emergence of novel PRRSv occurred in certain production systems, escalating to epidemic proportions when other, highly connected systems were reached. Sow farms seem to have a potential role in maintaining and spreading the disease within those production systems (27).

### Modeling

Although most PRRS outbreaks were reported in areas with the highest hog density, between farm PRRS transmission patterns differed substantially among regions. Differences in transmissibility were reported between different regions and systems, with evident peaks of disease occurring in different regions at different times. Results suggested that factors other than season and swine density might have a major impact on PRRS spread at the regional level, but such factors need to be elucidated (28).

### Prototype of a Real-time Data Visualization Dashboard

**Figure 1:**
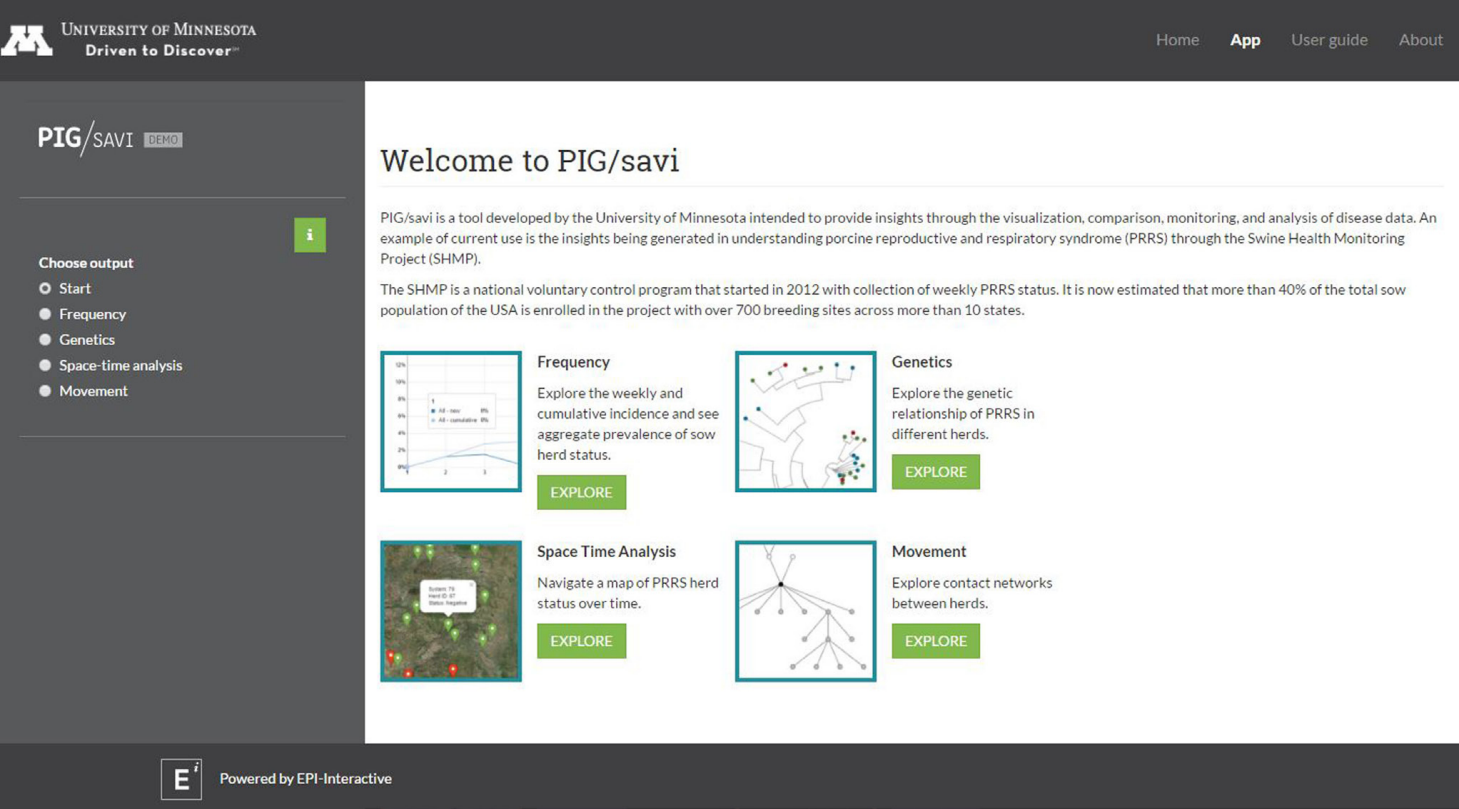
Dashboard of the RStudio Shiny-based application [Pig/Statistical Analysis and Visual Insights (SAVI)] with its four core analytical components: frequency, genetics, space time analysis, and movement.

The RStudio Shiny application platform integrates portions of the data described above into a system that allows near real-time data sharing, visualization, and analysis to support disease prevention and control. Its aim is also to improve the accessibility of the data and to add value to the program for different system stakeholders. The current array of tools developed as part of the epidemiological platform includes descriptive visualizations of prevalence and incidence over time, real-time risk maps, network visualizations, and molecular epidemiology tools, including the ability of the user to build different types of phylogenetic trees (Figure 1).

## Discussion

The MSHMP efforts resulted in a unique dataset generated through a voluntary system, which demonstrates U.S. producers’ commitment to sharing data toward the common goal of mitigating PRRS impact in the country. However, collection of data by itself has no value unless it is analyzed to bring knowledge and wisdom to improve speed and quality of decisions (30). Here, the value of sharing, aggregating data, and creating knowledge has been demonstrated with the use of multidisciplinary analytic tools that helped to maintain producer’s interest in a voluntary data sharing project. In the absence of a regulatory framework, data sharing is a prerequisite for increasing disease awareness and, ultimately, supporting its control. Lessons learnt from the systematic analysis of the MSHMP relate to the repetitive time and time–space patterns associated with PRRS incidence in the U.S. and the regional variation, and the influence that changes on epidemiological conditions, may have in those patterns. In addition, quantitative evidence was collected on how phylogenetic analysis may support investigations on the most likely mechanisms of spread of the virus, and how collaboration and data sharing may contribute to the control of the disease at the regional level in the absence of a regulatory framework.

A negative association between sharing PRRS status and incidence was found. It is unknown if this relationship is because negative farms are more inclined to share the data or if the increase in awareness has an effect on the incidence of the disease. However, in that specific study, the participation of farms, as well as PRRS status sharing, increased in the time frame studied suggesting that farmers and veterinarians may perceive collaboration as an advantage (25).

Noteworthy, even though a substantial amount of effort has been devoted to describing PRRSv transmission routes (15–18), many aspects of the disease epidemiology are yet-to-be-elucidated. For example, the proportional importance of transmission routes (air, pig movement, or fomites) in the spread of the disease and whether that pattern is mostly influenced by system or region remain unknown. The application of different analytical tools to the rich dataset assessed here has helped producers and practitioners to better understand the epidemiological dynamics of PRRS. For example, we learned that the onset of the disease had a repetitive pattern and a similar starting point from the first 4 years of data of the project, from 2009 to 2012 (23). However, data from 2013 and 2014, when PED emerged in the country, failed to signal the epidemics at the same date as predicted by previous years, showing a delay of 3 weeks (24). Also during that same season (2013 and 2014), the same cohort of farms showed a 50% decrease in the number of PRRS outbreaks between October and March when compared with the average observed in the same season in the previous four previous years. Many factors may have impacted the change in disease pattern observed that year, including an increase of biosecurity practices associated with PED emergence, an increase of PRRS vaccination, secular cycles, or difficulty performing the routine PRRSv diagnostics following high PED mortality rates. Furthermore, no changes in the centroid of the epidemic clusters were observed between 2009 and 2014, suggesting that although incidence decreased that year, the spatial dependence of the disease prevailed. Preliminary results of disaggregating national data and aggregating cases from different states (not published) seem to point to a different temporal incidence trend between geographical regions and/or systems, and those could be caused by different factors as management, density or different climate conditions as pointed by the ecological niche modeling (see text footnote 1).

Higher disease incidence was observed and linked to new PRRSv introduction that quickly disseminated in an area or system (27). Those observations were also supported by the description of PRRS transmissibility across regions and systems using the TD-R (28). Interestingly, the two densest swine regions showed different patterns of disease transmission, which may be somehow explained by a difference in management and PRRS immunization. The TD-R showed to be a valuable method to signal PRRS epidemics on those areas where outbreaks are relatively rare. For areas in which PRRSv is endemic, TD-R did not show any fluctuation and revolved around 1, which is expected for an endemic disease such as PRRS.

The finding that having crops or trees around the farm could influence the occurrence of new cases was intriguing and may have multiple interpretations and applications (26). It would be, indeed, interesting to assess whether the use of natural tree buffers could make a significant impact on the protection of the herd. To the best of our knowledge, there is no information on how vegetative buffers could prevent the spread of the diseases or protect the herd from PRRS infection. Similar conclusions were extracted using an ecological niche modeling approach in which land cover accounted for much of the variation in PRRS risk, after hog density and precipitation (see text footnote 1). However, further analysis is needed to confirm these findings.

Because sharing information was perceived by at least some companies as a competitive advantage as they can learn new insights on the disease epidemiology, some MSHMP participants started sharing PRRSv ORF5 sequences. ORF 5 sequencing has recently become a relatively common practice within the swine industry. The amount of available data from Veterinary Diagnostic Laboratories, and the use of new molecular epidemiology tools may help elucidate the relative importance of different transmission routes during outbreak investigations. For example, using ORF5 sequences from five different companies and Bayesian phylodynamic methods that could integrate time, space, and phylogenetic analysis allowed for a better estimate on the history of the virus, helping us to understand the underlying connections in the disease spread process (27).

As part of the same project, a platform that could provide access to data and analytical outputs in real time was also explored and developed. Such a platform could provide a decision advantage during the control of the disease and hence, potentially provide and demonstrate value of the MSHMP to the industry. Thus, an application was built using the R software and the R Study Shiny platform to link MSMHP outputs with different stakeholder groups. At this stage, the epidemiological tools included: descriptive epidemiology (incidence and prevalence over time), phylogenetic analysis, risk maps, and network analysis. Further extensions are possible. Having all the updated information in a real-time fashion can help people involved in health management to make better-informed decisions. It might also be used to replace labor-intensive scheduled reporting resulting in greater system efficiency and improve stakeholder engagement as previously demonstrated (31).

## Conclusion

The review here summarizes the knowledge gained over the last 6 years on the epidemiological dynamics of the most devastating disease affecting the U.S. swine industry through the voluntary MSHMP. When evaluating PRRS incidence data at a national scale, there is a predictable incidence pattern, with winter offering environmental conditions most favorable for virus spread at long (transport and fomites) and short (airborne) distances, and hog density and system being the most relevant factors associated with the spatial distribution of disease. However, when incidence data are explored at low levels of aggregation (state or county), factors such as presence of trees, being located in a hilly area, or fine-scale weather conditions are most influential on the risk for PRRS spread. Finally, this review demonstrates the level of contribution that the use of novel multidisciplinary analysis tools applied to routine collection of large surveillance data may provide to prevention and control of livestock diseases in the U.S. and at a global scale.

## Author Contributions

CV—primary author. AA, PM, UM, MA, PV-D, ST, RM, and AP—contributing authors. RM designed and supervised the data collection and storage process. AP designed and supervised the data analysis process. All the authors reviewed the manuscript and participated in the review process.

## Conflict of Interest Statement

The authors declare that the research was conducted in the absence of any commercial or financial relationships that could be construed as a potential conflict of interest.

## Footnotes

^1^Alkhamis M, Arruda AG, Vilalta C, Morrison R, Perez A. Surveillance of porcine reproductive and respiratory syndrome virus in the United States using risk mapping and species distribution modeling. *Prev Vet Med*.
